# Modelling prokaryote gene content

**Published:** 2007-02-05

**Authors:** Matthew Spencer, Edward Susko, Andrew J. Roger

**Affiliations:** 1 Department of Mathematics and Statistics, Dalhousie University, Halifax, Nova Scotia, Canada; 2 Department of Biochemistry and Molecular Biology, Dalhousie University, Halifax, Nova Scotia, Canada

**Keywords:** gene content, lateral transfer, phylogenetics, likelihood

## Abstract

The patchy distribution of genes across the prokaryotes may be caused by multiple gene losses or lateral transfer. Probabilistic models of gene gain and loss are needed to distinguish between these possibilities. Existing models allow only single genes to be gained and lost, despite the empirical evidence for multi-gene events. We compare birth-death models (currently the only widely-used models, in which only one gene can be gained or lost at a time) to blocks models (allowing gain and loss of multiple genes within a family). We analyze two pairs of genomes: two *E. coli* strains, and the distantly-related *Archaeoglobus fulgidus* (archaea) and *Bacillus subtilis* (gram positive bacteria). Blocks models describe the data much better than birth-death models. Our models suggest that lateral transfers of multiple genes from the same family are rare (although transfers of single genes are probably common). For both pairs, the estimated median time that a gene will remain in the genome is not much greater than the time separating the common ancestors of the archaea and bacteria. Deep phylogenetic reconstruction from sequence data will therefore depend on choosing genes likely to remain in the genome for a long time. Phylogenies based on the blocks model are more biologically plausible than phylogenies based on the birth-death model.

## Introduction

### The need for models of gene content

Some genes are patchily distributed over the prokaryote phylogeny. This pattern can be explained either by the presence of these genes in a common ancestor, followed by multiple gene losses, or by lateral transfer (Boucher et al. 2003). Here, we develop probabilistic models for the number of genes in gene families, and use these models to estimate the rates of gene loss and lateral transfer. Knowing these rates will help us to decide whether multiple gene loss or lateral transfer is a better explanation for the observed distribution of genes.

A gene family is defined as all the genes in a genome that belong to a group of repeated sequences (Graur and Li, 2000, p. 264). These sequences ultimately derive from a common ancestor, either by duplication or by lateral transfer from another lineage. All genes in a genome may have been descended from a single ancestral sequence in the distant past, so in practice, we (either implicitly or explicitly) choose a divergence time beyond which genes are placed in different families. One might divide the data into many small families to resolve close relationships, or few large families to resolve distant relationships. Different levels of family resolution do not always give compatible trees (Hughes et al. 2005), although this may be simply due to noise. Here, we use gene families from the COG (Clusters of Orthologous Groups of proteins) database (Tatusov et al. 2003). We chose the COG database because it has been used for several other gene-content-based phylogenies, allowing us to compare our results with those from other methods. In the COG algorithm, families are identified based on three-way sets of mutual best hits (Tatusov et al. 1997). Three-way best hits do not depend on the absolute similarity between sequences, which should reduce the effects of variation in evolutionary rates on gene family identification. However, this approach cannot identify gene families that occur in fewer than three genomes.

Previous attempts to estimate the rates of gene loss and lateral transfer have usually relied on parsimony analysis of the presence and absence of gene families. For example, Kunin and Ouzounis (2003) assigned ancestral gene states to internal nodes on a phylogeny, assuming equal penalties for gain and loss of genes. If the difference between the number of gains and losses required to explain the pattern of gene content in descendants of a node was larger than an arbitrary threshold, they assigned family presence to the node. If it was less than another arbitrary threshold, they assigned family absence to the node. Ambiguous cases were resolved by assuming that the ancestral state of every family was absence, and that the appearance of new families should be delayed as long as possible, moving from the root to the leaves of the tree. There are major problems with this approach. First, the number of events will be underestimated (Hao and Golding, 2004). If a gene family has the same state in two sister taxa, Kunin and Ouzounis (2003) assume that the family had this state in the common ancestor of the taxa. Cases of inparalogy *sensu* Sonnhammer and Koonin (2002), in which parallel gene gains occurred in sister taxa, will not be dealt with correctly. Second, the penalties for gain and loss cannot be estimated from the data, but must be fixed in advance. This is a major problem, given that these are the events we are interested in. Other parsimony-based methods have used different criteria for deciding on gain and loss penalties. For example, Mirkin et al. (2003) and Boussau et al. (2004) chose penalties that gave plausible metabolic pathways in reconstructed ancestors. Since there is no way to determine whether one set of penalties is significantly better than another for a given data set, we cannot make a choice between penalties chosen using different external criteria. Third, the thresholds for assigning family presence and absence to nodes are arbitrary. Kunin and Ouzounis (2003) used an ad-hoc method to estimate one of these thresholds, and fixed the other without reference to any data. Fourth, different ways of resolving ambiguous internal states give different results (Boussau et al. 2004), and the choice of one resolution is arbitrary. Fifth, this method ignores uncertainty in the assignments of internal states. Unobserved data on internal nodes are treated in exactly the same way as observed data in extant taxa. In reality, the probability of correct reconstruction can be low when the rates of change between states are large (Maddison, 1995).

All of these problems can be solved by using maximum likelihood methods with an explicit model for gene gain and loss. Under a likelihood approach, multiple and parallel changes are not ignored. If two sister taxa each have 10 members of a gene family, we do not assume that their common ancestor also had 10 members. If the two taxa are closely related, it is most likely that there have been few deletions and duplications, so the genes will probably be orthologs related by speciation. On the other hand, if the two taxa are distantly related, many independent changes may have occurred and the genes may be inparalogs *sensu* Sonnhammer and Koonin (2002). Gain and loss parameters are estimated from the data, rather than from arbitrary criteria. We can then use statistical methods to test hypotheses about these parameters. Uncertainty in internal states is not ignored. Instead, the likelihood is summed over ancestral states weighted by their probabilities.

There have been several attempts to develop explicit models for gene gain and loss. Huson and Steel (2004) derived a maximum likelihood distance for gene presence/absence data from a simple model for the number of genes in a genome. In their model, new genes are acquired at a constant rate, and existing genes are lost at a constant rate per gene, the same for all genes. They showed that both Dollo parsimony and their maximum likelihood distance outperformed the distance measure used by Snel et al. (1999) in phylogeny estimation from simulated data. It is not easy to estimate the rate of lateral transfer from presence/absence data, because we cannot distinguish between the evolution of a new gene and the acquisition of a gene by lateral transfer. However, if we assume that the evolution of new genes is rare, then maximum likelihood can be used with presence/absence data to estimate the rate of lateral transfer and gene loss (Brian Golding and Weilong Hao, McMaster University, personal communication).

Data on the number of members of a gene family could give more information on the rates of gene gains, losses and transfers. As far as we know, all existing models of gene content that count the number of members of gene families use birth-death models (Gu, 2000; Gu and Zhang, 2004; Zhang and Gu, 2004; Arvestad et al. 2003, Arvestad et al. 2004; Karev et al. 2004; Felsenstein, 2004; Hahn et al. 2005). In these models, the number of genes in a family can increase or decrease by the deletion or duplication of one gene at a time. This is unrealistic, because there is strong empirical evidence that multi-gene events can occur. For example, duplications of blocks of three to four genes (the typical operon size) are common in prokaryotes (Gevers et al. 2004). The γ-proteobacterium *Vibrio vulnificus* apparently gained 495 genes in 260 duplication events, when compared to its close relative *V. cholerae* (Chen et al. 2003). It is almost certainly possible to transfer or delete more than one gene at a time. In *E. coli*, the average gene is 1kb. Among closely related strains, the average acquired segment is 8.3 kb, and the average deletion is 6.4 kb (Ochman and Jones, 2000). Similarly, detected deletions among clinical clones of *Mycobacterium tuberculosis* contained an average of 4 open reading frames (Kato-Maeda et al. 2001). Detected insertions (apparently acquired by lateral transfer) contained between 2 and 36 open reading frames (ORFs), and detected deletions contained between 2 and 24 ORFs (Ochman and Jones, 2000). If the fixation of laterally transferred genes in local subpopulations depends on selection, and several genes are needed to perform a particular function, a transfer of all the required genes at once would be more likely to be fixed than a transfer of only one of the genes (Boucher et al. 2003, p. 319). Here, we compare the fit of a birth-death model to real data on pairs of prokaryote genomes with that of models allowing multi-gene events (blocks models). We show that blocks models are a better description of the data for two closely-related *E. coli* strains, and for the distantly-related pair *A. fulgidus* (archaea) and *B. subtilis* (gram-positive bacteria).

If genes may appear (by evolution from other sequences, duplication, or lateral transfer) and disappear (by deletion) from genomes, any single gene is unlikely to have been present in a lineage for its entire history. We can think of this using the ‘rope metaphor’ for a genome (Zhaxybayeva and Gogarten, 2004): there may be no single fiber (gene) running the entire length of the rope, yet the rope itself (the organismal lineage) is continuous. If we know the rates of appearance and disappearance of genes, we can estimate the expected residence time of a gene in the genome of a lineage. This tells us how far back in time we might be able to reconstruct the history of an organism from a single gene. We calculate residence times for the *E. coli* strains and for *A. fulgidus* and *B. subtilis*. We then use the estimated divergence times between pairs of taxa to estimate a least-squares phylogeny for 66 sequenced genomes in the COG database.

## Unobservable data

For nucleic acid or protein sequences, all states are observable in extant taxa. In contrast, a gene family that is absent from every taxon in the data set may not be observable. One way to solve this problem is to calculate likelihoods conditional on a gene family being observable (Gu, 2000). However, a gene family does not appear in the COG database unless it is present in at least three taxa. A gene family present in both members of a pair is not necessarily observable, and a gene family absent from both members of a pair is not necessarily unobservable. It is therefore difficult to use the conditioned likelihood approach when estimating pairwise maximum likelihood distances. Instead, we used a locally-weighted least-squares method to impute the numbers of unobserved gene families for each pair of taxa, as described in the methods.

## General form of the models

We make two key assumptions, family independence and a finite number of states, that allow us to model the dynamics of gene family size as a finite-state continuous-time Markov chain.

A realistic model for genome evolution would allow multigene events to affect members of more than one family, whenever genes from two or more families were adjacent on the genome (Felsenstein, 2004, page 515). Such a model would have to specify which genes are adjacent to each other, and would not treat each family independently. Functional relationships could also introduce dependencies, whether genes were adjacent or not. Both processes result in dependence across the entire genome. Thus, the number of possible states would be the number of different ordered genomes. When calculating the likelihood, we would have to sum over all possible states at the root (Equation 1, below). This becomes unfeasible as the number of possible states gets very large. Probabilistic models of gene order exist (Miklós, 2003) but are too complicated to use for phylogenetic inference. Adding changes in gene content will make this worse, as well as requiring more parameters and assumptions. Treating families as independent seems necessary if unrealistic. As far as we know, all existing probabilistic models for gene content make this assumption (Gu, 2000; Gu and Zhang, 2004; Arvestad et al. 2003, Arvestad et al. 2004). Fortunately, the consequences of assuming family independence are unlikely to be severe. Our models describe the dynamics of a single gene family. The parameter estimates therefore depend on the marginal distribution of gene family size within a family, not the joint distribution across gene families. This marginal distribution is unaffected by dependencies among families. We can think of dependencies among gene families as reducing the number of independent observations. Asymptotically, this will affect the variances but not the means of the parameter estimates. Since we have large numbers of data, the small-sample bias introduced by the incorrect variance is unlikely to be large. An analogous situation occurs in RNA evolution. Base pairing introduces strong dependence between sites, but treating sites as independent does not significantly reduce the accuracy of phylogeny estimation by likelihood (Tillier and Collins, 1995).

In principle, the number of genes in a family can take any non-negative integer value. Nevertheless, most gene families have only a few representatives in a given genome (Huynen and van Nimwegen, 1998). To simplify the calculation of likelihoods, we will use *k* + 1 states, corresponding to [0, 1, 2, 3,… *k* − 1, ≥ *k*] genes. We will make *k* large enough that the *k* th state is rarely reached, so the errors due to truncation will be small. Over the whole COG database, there were 83675 cases where a gene family was present in a species. Of these, only 320 (0.4%) were cases of families with more than 20 members. We therefore choose *k* = 20. We also did preliminary experiments using *k* = 10, with similar results.

Given these two assumptions, our models can be based on a matrix **Q** of instantaneous rates of transition *q_ij_* from state *i* to state *j*. We use a homogeneous model, in which **Q** is the same everywhere, because non-homogeneous models require many more parameters. We have only two taxa and their common ancestor, so there is only one tree topology to consider. The likelihood *L* of the data for a given model is

(1) $$L=\prod_{n=1}^{N}\sum_{i=0}^{k}\pi_{i}P(i,j_{1}(n)|t_{1})\;P\;(i,j_{2}(n)|t_{2})$$

where *N* is the number of families, π_i_ is the stationary probability of the *i* th state, *j*_s_(*n*) is the state of the *n* th family in genome *s* = {1, 2}, and *t*_s_ is the time separating genome *s* from the common ancestor of the two genomes. The transition probabilities *P*(*i, j_s_* (*n*) | *t_s_*) from state *i* to state *j* in genome *s* time *t_s_* are given by the matrix

(2) $$P(t_{s})=e^{Qt_{s}}=\sum_{m=0}^{\infty }\frac{{\left(Q\;t_{s}\right)}^{m}}{m!}$$

(Norris, 1997, pp. 62–63). Because we cannot obtain absolute rates, we express all the *q*_ij_ relative to *q*_01_, and scale the **Q** matrix so that the expected number of events per gene family per unit time is 1 (Yang, 1994a). The times *t_s_* are then expected numbers of events per gene family.

**Figure 1:**
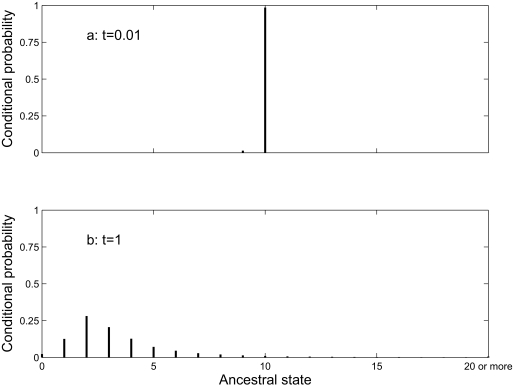
Conditional probabilities of each possible ancestral state, given 10 members of a gene family in two taxa, each separated from a common ancestor by an edge of length 0.01 (a) or 1 (b) expected changes. Calculated under the blocks model with parameters (other than edge lengths) from Table 3.

In Equation 1, the summation is over all possible ancestral states *i*. This allows us to deal correctly with inparalogy. Figure 1 illustrates this for a single gene family in a hypothetical case (using the blocks model described below, with parameters other than edge lengths from Table 3). Two sister taxa, each with 10 genes in the family, are connected to a common ancestor by edges of length *t*. The conditional probability of an ancestral state *h* is π*_h_* *P*(*h,*10|*t*)*P* (*h,*10|*t*)/*∑_i_*_=0_*^k^* π_i_*P*(*i,*10|*t*) *P*(*i,*10|*t*) When *t* is small (Figure 1a), the conditional probability that the common ancestor also had 10 genes is 0.985. The genes are most likely to be orthologs, each being related by speciation. When *t* is large (Figure 1b), the distribution of conditional probabilities is much broader. The conditional probability of 10 genes in the common ancestor is very small (0.011). Ancestral states with few genes have higher conditional probabilities, so most of the observed genes are likely to be inparalogs that arose by parallel gains. For these data, parsimony-based methods (Kunin and Ouzounis, 2003; Hao and Golding, 2004; Mirkin et al. 2003; Boussau et al. 2004) always assign 10 genes to the common ancestor. When the divergence time is large, this will be a poor choice.

The processes by which gene content may change are deletion, duplication, lateral transfer, and innovation (the evolution of a new member of a gene family from an existing sequence). Since the set of taxa under study is much smaller than the set that could have given rise to transfers, we assume that lateral transfers always come from a taxon other than the pair of taxa we are analyzing. We now consider some specific models.

## Birth-death models

In a birth-death model, we assume that one gene can be duplicated or deleted at a time. In the standard form of this model (Kendall, 1948), we assume that each gene has independent and equal probabilities of deletion (*f*) and duplication (*g*). Thus

(3) $$q_{ij}=\{\begin{array}{ll} g_{i} & j=i+1,i<k\\ f_{i} & j=i-1,i>0\\ 0 & \text{otherwise} \end{array}$$

Note that the state space here is finite, with states [0, 1,…, *k* − 1, ≥ *k*], unlike the infinite-state model described in Kendall (1948). In equation 3, the zero state is absorbing, so the stationary probability has all its mass at zero. When calculating the likelihood of molecular phylogenetic data given a tree and parameter estimates, it is usual to assume a stationary distribution of states at the root (equation 1) (Felsenstein, 1981). If we do this for the model in equation 3, any pattern other than zeros at every leaf has probability zero. We could assume that at least one member of the family was present at the root (Gu, 2000; Gu and Zhang, 2004; Zhang and Gu, 2004; Arvestad et al. 2003, Arvestad et al. 2004). Alternatively, we could add a non-zero rate from state zero to state one, so that zero is no longer an absorbing state (Karev et al. 2002). This rate represents either lateral transfer or innovation. Huson and Steel (2004) used a model with a constant birth rate for all states, which has similar consequences.

We will use a model called the linear birth-death-innovation model by Karev et al. (2002), in which there is a non-zero rate *e* from state zero to state one, and the other single-gene events have both linear (*gi* for duplications, *fi* for deletions from state *i*) and constant (*g*_2_ and *f*_2_) components. This is a generalization of the standard birth-death model and of the model used by Huson and Steel (2004). More complex models with other kinds of relationship between gene family size and rates have been considered (Karev et al. 2003, Karev et al. 2004). As in all our models, we will truncate at an upper limit state *k*. This model is time-reversible, so the two edge lengths *t*_1_ and *t*_2_ affect the likelihood only through their sum. Table 1 illustrates the structure of the **Q** matrix for this model. Non-zero values of the constant terms *f*_2_ and *g*_2_ are evidence for processes other than independent deletion and duplication of single genes, including lateral transfer. In future, we will refer to this model simply as birth-death.

## Blocks models

In a blocks model, we divide the **Q** matrix into blocks representing different kinds of process, and assume that duplications, deletions and transfers can affect multiple genes within a family. As in the birth-death model, we assume that duplication and deletion events operate independently and at a constant rate on each possible unit. In the blocks models, the units may be larger than one gene. If the order of genes on the genome is unimportant, then the number of possible units on which an event can operate to cause a transition from state *i* to state *j* is $\left(\begin{array}{c} i\\ \left|j-i\right| \end{array}\right)$. We assume that each existing gene is equally likely to be part of a unit. We model innovation, deletions and duplications of single genes exactly as in the birth-death model, with the exception of the transition from state 1 to state 0. For deletions that result in the loss of an entire gene family, there is only one possible unit (the whole family). We therefore assign a constant rate *a* to the transition from all states *i* > 1 to 0. We allow a separate parameter for these deletions because loss of an entire family might have different consequences from loss of some but not all genes in a family. We assign a separate parameter *h* to the transition from 1 to 0. We model the rates of deletion of more than one but less than an entire family of genes as $\left(\begin{array}{c} i\\ \left|j-i\right| \end{array}\right)b+b_{2}$. Similarly, we model the rates of duplications of more than one gene as $\left(\begin{array}{c} i\\ \left|j-i\right| \end{array}\right)c+c_{2}$.

We model transitions from state *i* to state *j* > 2*i* with a constant rate *d*. These transitions correspond only to lateral transfers. Duplication cannot cause a transition from *i* to state *j* > 2*i*. Innovation can create new members of an existing family, but cannot create more than one new member at a time. For example, if a non-coding sequence is duplicated and both copies become genes soon after by acquiring start codons, they will probably both be placed in the same family, but two separate mutations at different times are required to create the start codons. We use a constant lateral transfer rate because we do not have much information on the distribution of the number of genes transferred from another genome in a single event. Table 2 shows an example **Q** matrix for a blocks model. This model is not time-reversible in general.

For the blocks model, *d* provides a direct estimate of the rate of acquisition of more than one member of a family by lateral transfer, when we are acquiring more genes than could be added by duplication or innovation.

## Model comparison

The blocks model has 13 parameters. The birth-death model has only five, and can be obtained from the blocks model by setting *a*, *b*, *b*_2_, *c*, *c*_2_ and *d* to zero, and *h* to *f* + *f*_2_. The blocks model can never be a worse description of the data than the birth-death model. We therefore need to establish that the improved fit of the blocks model is statistically significant. To do this, we use likelihood-ratio tests (for a review of likelihood ratio tests in phylogenetics, see Huelsenbeck and Crandall, 1997).

Two models are nested when the simpler model is a special case of the more complex one (as in our situation). If the simpler model is the true model, the extra parameters in the more complex model are unnecessary and will give only a small improvement in fit. In most such cases, twice the log likelihood ratio has a distribution approaching χ*_n_*^2^, for a pair of models in which the values of *n* parameters are fixed in the simpler model. Thus, if twice the observed log likelihood ratio was unlikely to have been drawn from the χ*_n_*^2^ distribution, we can reject the hypothesis that the extra parameters did not significantly improve the fit. Setting parameters to boundary values such as zero involves minor changes to this approach, as described in the appendix. We are also interested in whether the estimated rate of lateral transfer in the blocks model is significantly greater than zero. As described in the appendix, we can test whether *d* > 0 using a likelihood-ratio test, although this will not tell us about transfers of one or a few genes from the same family. If the blocks model is significantly better than the birth-death model, it will be useful to know which parameters are important. We therefore use likelihood-ratio tests to compare the full blocks model to models with no multiple gene losses (*a, b* and *b*_2_ set to zero) or no multiple gene gains (*c*, *c*_2_ and *d* set to zero).

The number of parameters we are estimating is not large, given that we have 4873 gene families, each with 21 possible states. For comparison, the General Time-Reversible model of nucleotide substitution has 8 parameters plus edge lengths, but these must be estimated from data with only four states. Amino acid substitution matrices typically require 189 parameters to model 20 possible states, and require very large databases for reliable estimation. It is often the case that even complex models do not fit phylogenetic data well, but this is not a major problem (Huelsenbeck and Crandall, 1997). We are interested in knowing whether a more complex model is a significantly better description of the data, even though we cannot capture all the details of the evolutionary process.

## Results

### *E. coli* data

**Figure 2:**
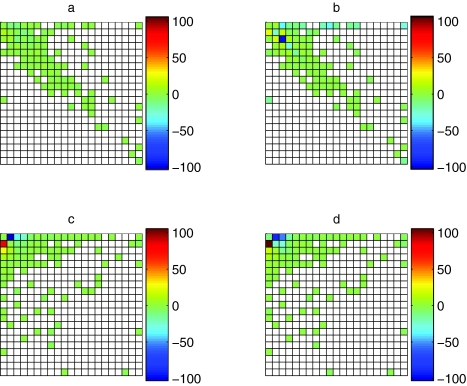
Performance of blocks and birth-death models for two *E. coli* strains K12 and 0157:H7 EDL933 (a: blocks model, b: birth-death model) and for *Archaeoglobus fulgidus* and *Bacillus subtilis* (c: blocks model, d: birth-death model). The data are *n̂_ij_* (log *f̂_ij_* _(model)_ − log (*n̂_ij_*/*n*)), the contribution to the log likelihood ratio between a given model and the best possible model from each pattern. *n̂_ij_* is the LOWESS imputed count of state *i* (row) in the first species and state *j* (column) in the second species, *f̂* *_ij_* _(model)_ is the model predicted relative frequency of pattern *ij*, and *n̂_ij_*/*n* is the LOWESS imputed relative frequency. States are ordered from 0 to ≥20 family members in both rows and columns. Cells are red where the model predicts too high a frequency and blue where it predicts too low a frequency. White cells are patterns for which there were no observations (these make no contribution to the likelihood).

For the *E. coli* data, the blocks model was much better than the birth-death model (Table 3, −2Δlog *L* = 522, conservative test using χ_8_^2^, *p* ≤10^−16^). The relatively poor fit of the birth-death model was mainly due to under-prediction of gene families absent from the K12 strain but with more than one member in the 0157:H7 EDL933 strain (first row, third and higher columns in Figure 2b), and in families having small and equal numbers of genes in both strains (the second to fifth entries of the main diagonal in Figure 2b). In contrast, the blocks model made good predictions for almost all patterns (Figure 2a).

In the blocks model, the estimated rate of lateral transfer of more genes than could be acquired by duplication or innovation ( *d* ) was three orders of magnitude lower than the estimated rate of acquisition of new gene families (*e*). Setting *d* to zero did not significantly reduce the likelihood (−2Δlog *L* = 1.10, tested against $\frac{1}{2}\chi_{0}^{2}+\frac{1}{2}\chi_{1}^{2}$, *p* = 0.15). However, setting all multiple gene gains to zero (*c*, *c*_2_ and *d* = 0, −2Δlog L = 18.07, conservative test using χ_3_^2^, *p* = 4 × 10^−4^) resulted in a significantly worse model. Setting all multiple gene losses to zero had an even stronger effect on the likelihood (*a*, *b* and *b*_2_=0, −2Δlog *L*=161, conservative test using χ_3_^2^, *p* ≤10^−16^).

**Figure 3:**
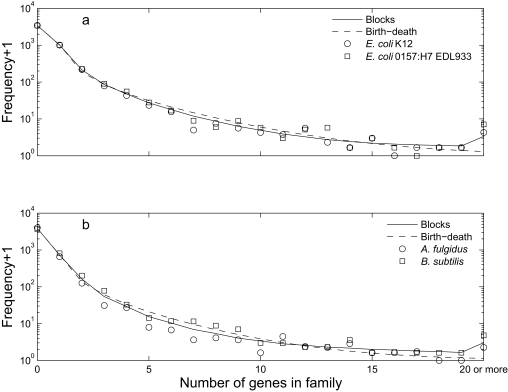
Marginal distributions of gene family size for single species. Symbols are the imputed counts used as data, and lines are predictions from the stationary distributions of the models, with parameters estimated from pairs of species. a: *E. coli* strains K12 (circles) and 0157: H7 EDL933 (squares), b: *Archaeoglobus fulgidus* (circles) and *Bacillus subtilis* (squares). In both panels, the blocks model is the solid line and the birth-death model is the dashed line. The vertical axis is on a logarithmic scale, so we use (frequency+1) to allow zero frequencies to be represented.

For both models, the predicted marginal distributions of gene family size in single species were quite close to the observed distributions (Figure 3a). For the *E. coli* data, the expected residence time of a gene was 0.60 under the blocks model. The distribution was strongly right-skewed, with a median of 0.33, a maximum of 9.06, a 95th percentile of 2.13 and a standard deviation of 0.76. 91% of simulated genes had residence times greater than the mean of *t*_1_ and *t*_2_ (0.04, the mean number of events separating one of the two strains from their common ancestor).

### *A. fulgidus* and *B. subtilis* data

For *A. fulgidus* and *B. subtilis,* the blocks model was significantly better than the birth-death model (Table 4, −2Δlog *L* = 74, conservative test using χ_8_^2^, *p* = 6 × 10^−13^). Both the blocks model (Figure 2c) and the birth-death model (Figure 2d) performed badly for patterns with small numbers of genes in both taxa, tending to overpredict the frequency with which gene families were absent from *B. subtilis* and present in *A. fulgidus* (first column, second and third rows of Figure 2c and d), and to underpredict the opposite case (first row, second to fourth columns of Figure 2c and d). The birth-death model also underpredicted the frequency of families with small but equal numbers of members in both taxa (Figure 2d, first three entries on main diagonal). These problems are due to the assumption of a homogeneous model, which is unlikely to hold over large evolutionary distances. For both models, the predicted marginal distributions (Figure 3b) are averaging over the small *A. fulgidus* and the large *B. subtilis* genome, but nevertheless have approximately the right shape. This suggests that it is much easier to build a plausible model for the distribution of gene family size in a single taxon than for sets of related taxa.

In the blocks model, *d* was seven orders of magnitude lower than *e*, and could be set to zero without significantly reducing the likelihood (likelihoods were the same to four decimal places). As with the *E. coli* data, models without any multiple gene losses (−2Δlog L = 13.71, conservative test using χ_3_^2^, *p* = 3 × 10^−3^) and gains (−2Δlog *L* = 25.56, conservative test using χ_3_^2^, p = 5 × 10^−4^) were significantly worse than the full model.

For *A. fulgidus and B. subtilis,* the expected residence time was 0.48 under the blocks model. Again, the distribution was right-skewed (median 0.34, maximum 3.71, 95th percentile 1.44, standard deviation 0.48). 42% of simulated genes had residence times greater than the mean of *t*_1_ and *t*_2_ (0.43 for these species).

### Model comparisons over all pairs of taxa

Over all 2145 pairs of taxa from the COG database, 95% had −2Δlog *L* ≥9. The median −2Δlog *L* was 85, and the range was 0 to 522. Thus, although the *E. coli* pair had the largest likelihood ratio (by chance: we did not know this in advance), the blocks model was substantially better than the birth-death model for most pairs.

### Phylogenies

**Figure 4:**
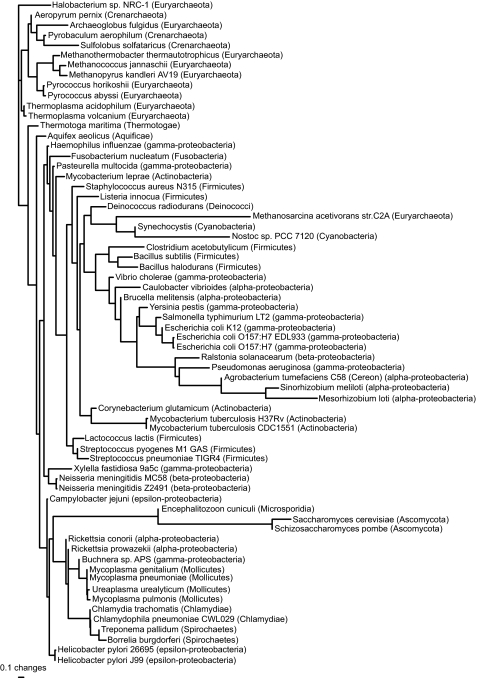
Phylogeny based on birth-death distances for all 66 genomes in the COG database, estimated by least squares with inverse square weighting (three equally good topologies were found, but they differed only in the arrangement of clades separated by zero-length edges). The tree is rooted with all the archaea except *Methanosarcina acetivorans* as an outgroup. Edge lengths are expected numbers of gene events per gene family. The weighted sum of squares was 830.

**Figure 5:**
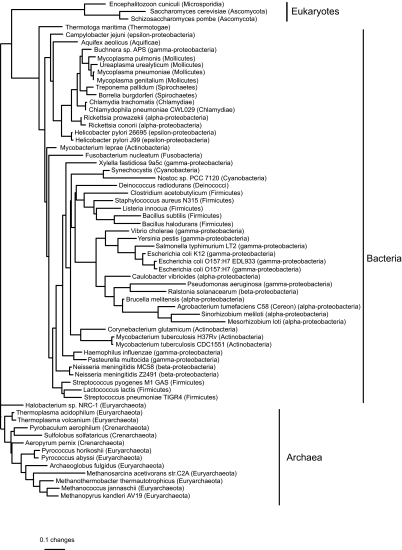
Phylogeny based on blocks model distances for all 66 genomes in the COG database, estimated by least-squares with inverse square weighting. The tree is rooted with the archaea as an outgroup. Edge lengths are expected numbers of gene events per gene family (note the difference in scale from Figure 4). The weighted sum of squares was 157.

We found three equally good least-squares trees based on distances from the birth-death model, but the only difference between them was in the arrangement of subgroups around zero-length edges within a clade containing parasitic/endosymbiotic α– and γ-proteobacteria, chlamydiae, spirochaetes, and mycoplasmas. The tree (Figure 4) is biologically implausible, placing *Methanosarcina acetivorans* (Archaea) with the cyanobacteria. *M. acetivorans* might be misplaced because it has an unusually large genome for an archaeon, perhaps due to the presence of retrointrons transferred from bacteria (Rest and Mindell, 2003). The three eukaryotes (*Encephalitozoon cuniculi, Saccharomyces cerevisiae, and Schizosaccharomyces pombe*) form a clade within the bacteria, perhaps because of mitochondrial genes transferred to the nucleus. Given these obvious problems, we do not discuss the trees from the birth-death model further. In contrast, the single best least-squares tree based on distances from the blocks model (Figure 5) has each of the three kingdoms as a monophyletic group.

Two other published phylogenies have been based on subsets of the same data: Wolf et al. (2002) used pairwise Jaccard distances among the 58 genomes then available, while Gu and Zhang (2004) selected 35 genomes and calculated distances based on a birth-death model. Like our tree from the blocks model, both have a clade containing parasitic and endosymbiotic bacteria with small genomes: the parasitic α-proteobacteria *Rickettsia spp.,* chlamydiae (*Chlamydia trachomatis, Chlamydophila pneumoniae*), spirochaetes (*Treponema pallidum and Borrelia burgdorferi*), mycoplasmas (*Mycoplasma spp.* and *Ureaplasma urealyticum*), and the endosymbiont γ-proteobacterium *Buchnera.* This clade is probably an artefact resulting from parallel loss of genes that are unnecessary for parasites (Wolf et al. 2001). Dealing with this artefact may require mixture models, in which different subsets of the data have different edge lengths.

## Discussion

There are many fundamental improvements that can be made to models of gene content, such as including gene order (Miklós, 2003) or nucleotide substitions (Arvestad et al. 2004). Unfortunately, these are usually very difficult. Here, we have shown that large improvements in likelihood over the widely-used birth-death models can be achieved by allowing multi-gene events, a change that is both simple and biologically reasonable. Our analyses show strong evidence that duplications and deletions of multiple genes are possible. This is not surprising, since we know that rearrangements of multiple genes occur in nature (Deng et al. 2003). The physical arrangement of the *E. coli* genome also suggests multi-gene events have occurred. For example, *E. coli* 0157:H7 EDL933 has two identical copies of a 106-gene island (Perna et al. 2001), which are unlikely to have arisen by repeated single duplications. Since the birth-death model does not allow multi-gene events, it is unlikely to be the best model for the evolution of gene content. This has been suggested elsewhere (Hahn et al. 2005), but we are not aware of any other models that address the problem. We assumed that the number of units on which events can operate to produce a transition from state *i* to state *j* is $\left(\begin{array}{c} i\\ \left|j-i\right| \end{array}\right)$. This ignores the order of genes on the genome. It might be possible to improve the model using a suitable distribution of breakpoint locations (Nadeau and Taylor, 1984; Pevzner and Tesler, 2003), so that duplications of few genes are more frequent than duplications of many genes. On the other hand, if genome rearrangements are so frequent that the order of genes is randomized between duplication or deletion events, our simple model might be a better choice.

For both pairs of taxa that we studied in detail, the estimated rate of lateral transfer of more genes from a single family than could be acquired by duplication or innovation was not significantly greater than zero. There is other evidence of lateral transfers into *E. coli.* For example, of 108 islands larger than 1kb present in *E. coli* 0157:H7 EDL933 but absent in *E. coli* K12, at least 84% had atypical base composition, which may suggest they are recent lateral transfers (Perna et al. 2001). Nevertheless, these events might only rarely transfer multiple genes from the same family. Alternatively, our assumption of a constant rate for all numbers of transferred genes might be unrealistic. The distribution of detected additions in *E. coli* strains has its mode in the 5kb (approximately 5-gene) category, with much lower rates for longer sections of DNA (Ochman and Jones, 2000, their figure 2). With a single rate for all numbers of transferred genes, we might underestimate the rate of transfer of small numbers of genes. Our models do not allow us to estimate the rate of lateral transfers of single genes, because we cannot separate such events from duplications and innovations. Nevertheless, the transition rate *e* from state 0 to state 1 was not negligible relative to other transitions (in both cases, about 1/5 of the rate of deletion of entire gene families). This transition can only happen by innovation or lateral transfer. If we believe that innovations are rare events, then *e* not being negligible is consistent with the widely-held view that lateral transfers of single genes are frequent. Brian Golding and Weilong Hao (McMaster University, personal communication) analyzed presence/absence data for closely related species of bacteria using conditional maximum likelihood on a known tree. They also found that the rates of gain and loss of gene families were substantial. However, most gene family sizes do not vary greatly among strains within species (Pushker et al. 2004), so the rate of lateral gene transfer may not be so high that vertical inheritance and gene duplication are unimportant.

The estimated edge lengths for *A. fulgidus* and *B. subtilis* under the blocks models were an order of magnitude greater than for the *E. coli* strains. This is expected as *A. fulgidus* and *B. subtilis* are from different kingdoms. More interestingly, the sum of edge lengths increased more under the birth-death model for *A. fulgidus* and *B. subtilis* than for the *E. coli* strains. When only single-gene events are allowed (as in the birth-death model), a large number of events are required to explain families with multiple members in one taxon and no members in another. As a result, the sum of edge lengths was much greater for the birth-death tree for all taxa (30 expected events per gene family) than for the blocks model tree (9 expected events per gene family). For both models, the poor prediction of families present in only one of *A. fulgidus* and *B. subtilis* suggests that the evolutionary process might not be homogeneous over large time intervals. This is supported by the fact that we only found optimization problems for pairs of taxa from different kingdoms. It is difficult to construct non-homogeneous phylogenetic models. In principle, we could have different rate parameters on every edge (Galtier et al. 1999), but we would need very large numbers of data to get good estimates. If we can identify groups of taxa (such as archaea and bacteria) with different rates *a priori*, we could have a small number of different rate matrices. Distances based on the logdet transformation do not require the assumption of homogeneity, and have been applied to gene content data (Lake and Rivera, 2004; Rivera and Lake, 2004), but do not provide parameter estimates for rates of gene gain and loss.

For birth-death-innovation models allowing only single-gene events, the mean time required for formation of the largest observed gene families is unrealistically high compared to the time available for the evolution of life (Karev et al. 2003, Karev et al. 2004). These authors suggest that the minimum rather than the mean time necessary for evolution of the largest observed gene families is the relevant statistic, and that the minimum time may be realistic for some models with nonlinear relationships between rates and family sizes. Another explanation is that multi-gene events have been important in the formation of large gene families.

For both pairs of taxa that we studied in detail, the median residence time for a gene under the blocks model was around 0.33 gene events. The distance between the last common ancestor of the bacteria and the last common ancestor of the archaea on the blocks model tree was 0.19 gene events. Furthermore, extant taxa are typically quite far from either of these last common ancestors. For example, the maximum likelihood distance between *A. fulgidus* and *B. subtilis* under the blocks model was about 0.4 gene events. Deep phylogenetic reconstruction from sequence data therefore depends on picking genes with long residence times, rather than using a large number of randomly selected genes. Phylogeneticists already do this to some extent, because genes with short residence times are less likely to be present in every sampled taxon than genes with long residence times. Adding rate variation among gene families to the blocks models would allow more accurate residence time calculation, and would help to identify families with long residence times. It is likely that these families will include the information transfer and central metabolic genes often thought of as the core genes (Lawrence and Hendrickson, 2003). Rate variation among gene families will also be important to deal with selection for large numbers of genes in some families.

Our phylogeny based on evolutionary distances from the blocks model showed many similarities to phylogenies based on distances from a birth-death model (Gu and Zhang, 2004) and from Jaccard distances (Wolf et al. 2002). Despite not being based on an evolutionary model, the Jaccard distance tree did not appear less biologically reasonable. All three trees shared a common weakness: parasites and endosymbionts with reduced genomes tended to be grouped together. This is probably a consequence of parallel evolution. The same genes are likely to be lost in many parasites and endosymbionts, because of the similar environments they inhabit. Such patterns require models that allow evolutionary rates to vary in different ways over time as well as between gene families: the genome equivalent of heterotachy (Lopez et al. 2002). Interestingly, trees based on the distances used in the SHOT web server (Korbel et al. 2002) do not have a problem with the placement of parasites and endosymbionts. This is because the distance measure used in SHOT assumes the shared absence of a gene is uninformative (which is probably true for parasites but not for other organisms). Methods that use normalized BLASTP scores, which take sequence similarity into account, also place the parasites and endosymbionts correctly (Gophna et al. 2005; Kunin et al. 2005). The good performance of SHOT distances and normalized BLASTP scores, which are not proper measures of evolutionary distance, suggests that the phylogenetic signal in whole genome data is far from saturation. A good modelling approach might therefore be to estimate a phylogeny using one of these simple methods, then fit more complex models on this phylogeny to estimate rates of gene gain and loss.

All three gene content trees (Wolf et al. 2002; Gu and Zhang, 2004, and our tree) misplaced *Halobacterium*, an archaeal species with many genes of bacterial origin. Two of the three trees (Wolf et al. 2002, and our tree) misplaced *Thermotoga*, a hyperthermophilic bacterium with many archaeal genes. It has been suggested that proper models of genomic evolution will be unaffected by lateral gene transfer (Lake and Rivera, 2004). We do not think this is true, because a taxon that acquired many genes by lateral transfer from the same source will tend to share the presence of genes with close relatives of that source.

Our tree based on the birth-death model was much less plausible than the birth-death-based tree of Gu and Zhang (2004). One possible explanation is that we modelled data on the number of members of each gene family, while Gu and Zhang (2004) modelled only absence, presence of one member, or presence of more than one member. It may be that the number of members of a gene family is not usually known accurately, and that aggregating into categories reduces the effect of unreliable data (Zhang and Gu, 2004). The way in which gene families are identified can also affect our ability to infer phylogenies. Hughes et al. (2005) showed that with presence-absence data, more stringent homology criteria for gene family identification gave better resolution for closely-related taxa, but retained less information about deep divergences. They also found that trees inferred from data with different homology criteria were not compatible, although this might be due to artefacts of parsimony.

In principle, we could use the blocks model for full maximum likelihood phylogenetic inference. One major benefit from this is that we might be able to allow different rates of evolution for different gene families. Rates-across-sites models typically give large improvements in likelihood for sequence data (Yang, 1994b), but cannot be identified from pairwise data alone. The blocks model is not reversible, so we would infer a rooted phylogeny. In practice, we expect estimation of the root location to be fairly inaccurate, as is usually the case for irreversible models of nucleotide evolution (Yang, 1994a; Huelsenbeck et al. 2002). A major difficulty with phylogeny estimation from gene content is that the absence of a family from the entire data set is not observable (Gu, 2000; Lake and Rivera, 2004). We used imputed counts to overcome this difficulty. Another approach is to condition on the probability that a family was present in at least three genomes, but calculating this probability would be very time-consuming. As far as we know, full maximum likelihood methods have only been used for gene content with very small sets of taxa (Zhang and Gu, 2004), and under a simplified conditional likelihood in which a gene family from the COG database was assumed to be observable only if present in at least one genome from the four under study.

### Conclusions

We have demonstrated that multi-gene events are an important component of genome evolution. Our models have the potential to reveal the relative importance of gene loss and lateral transfer, can provide estimates of the rate of turnover in genomes, and may lead to improved phylogenetic estimation from gene content data.

## Figures and Tables

**Figure 6:**
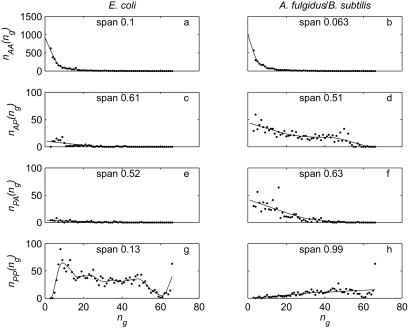
Relationship between number of genomes in which a gene family is found (horizontal axis, *n_g_*) and number of observations of a category in the focal pair of genomes (vertical axis, *n*..(*n_g_*)), where *n*.. is one of the categories *AA* (a, b), *AP* (c, d), *PA* (e, f) and *PP* (g, h). *A* indicates absent and *P* present in each member of the focal pair. Focal pairs are *E. coli* strains K12 and 0157:H7 EDL933 (a, c, e, g); *Archaeoglobus fulgidus* and *Bacillus subtilis* (b, d, f, h). Dots are observations, and solid lines are LOWESS curves with span (proportion of points used in each local regression) indicated on each panel. The vertical axis scale is fifteen times larger in a and b than in the other panels.

**Figure 7:**
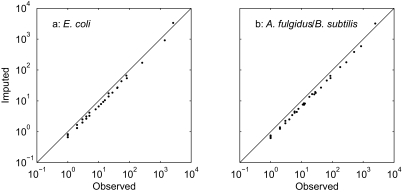
Double logarithmic plots of observed (*n_kl_*) versus imputed counts (*n̂_kl_*) for (a) *E. coli* strains K12 and 0157:H7 EDL933, and (b) *Archaeoglobus fulgidus* and *Bacillus subtilis*. The line indicates equality. The upper right-hand point is the (0, 0) pattern (absent from both members of the pair) for both pairs of taxa.

**Table 1:** Structure of the instantaneous rate matrix **Q** for the linear birth-death-innovation model, illustrated with *k* = 6. Diagonal elements (not shown) are −∑*_i_*_≠_*_j_* *q_ij_*. The states (labelled in the first row and column) are 0,1…k−1, ≥ k members of a gene family.

	0	1	2	3	4	5	≥6
0	-	*e*	0	0	0	0	0
1	*f* + *f*_2_	-	*g* + *g*_2_	0	0	0	0
2	0	2*f* + *f*_2_	-	2*g* + *g*_2_	0	0	0
3	0	0	3*f* + *f*_2_	-	3*g* + *g*_2_	0	0
4	0	0	0	4*f* + *f*_2_	-	4*g* + *g*_2_	0
5	0	0	0	0	5*f* + *f*_2_	-	5*g* + *g*_2_
≥6	0	0	0	0	0	6*f* + *f*_2_	-

**Table 2:** Structure of the instantaneous rate matrix **Q** for the blocks model, illustrated with k = 6. Diagonal elements (not shown) are −∑_i≠j_ q_ij._ The states (labelled in the first row and column) are 0,1…k − 1, ≥ k members of a gene family.

	0	1	2	3	4	5	≥6
0	*-*	*e*	*d*	*d*	*d*	*d*	*d*
1	*h*	*-*	*g + g2*	*d*	*d*	*d*	*d*
2	*a*	2*f* + *f*_2_	*-*	2*g* + *g*_2_	*c* + *c*_2_	*d*	*d*
3	*a*	3*b* + *b*_2_	3*f* + *f*_2_	*-*	3*g* + *g*_2_	3*c* + *c*_2_	*c* + *c*_2_
4	*a*	4*b* + *b*_2_	6*b* + *b*_2_	4*f* + *f*_2_	*-*	4*g* + *g*_2_	6*c* + *c*_2_
5	*a*	5*b* + *b*_2_	10*b* + *b*_2_	10b + *b*_2_	5*f* + *f*_2_	*-*	5*g* + *g*_2_
≥6	*a*	6*b* + *b*_2_	15*b* + *b*_2_	20b + *b*_2_	15*b* + *b*_2_	6*f* + *f*_2_	*-*

**Table 3:** Parameter estimates for *E. coli* strains K12 and 0157:H7 EDL933. **Q** matrix parameters are scaled so that the expected number of events per unit time is 1, and edge lengths *t*_1_ (from the root to K12) and *t*_2_ (from the root to 0157:H7 EDL933) are in expected numbers of events per gene family. Parameters that were constrained to be zero are indicated by -. In the birth-death model, *h* was constrained to be *f* + *f*_2_.

model	blocks	blocks, d = 0	no multi-gene gains	no multi-gene losses	birth-death
log likelihood	−5.8975 × 10^3^	−5.8980×10^3^	−5.9065×10^3^	−5.9779e + 03	−6.1584 × 10^3^
*a*	1.11	1.20	1.21	-	-
*b*	2.71×10^−27^	3.15×10^−39^	3.29×10^−29^	-	-
*b*_2_	0.62	0.53	0.42	-	-
*c*	3.06×10^−4^	3.22×10^−4^	-	6.97 × 10^−5^	-
*c*_2_	0.20	0.24	-	0.05	-
*d*	5.21×10^−4^	-	-	6.31 × 10^−^4	-
*e*	0.27	0.27	0.26	0.18	0.11
*f*	3.95×10^−5^	2.51×10^−7^	8.86×10^−21^	1.28	1.66
*f*_2_	0.27	0.11	0.07	6.20 × 10^−5^	−1.29
*g*	1.22	1.26	1.66	0.48	1.35
*g*_2_	−0.60	−0.61	−1.04	0.05	−0.98
*h*	0.47	0.42	0.37	0.64	*f* + *f*_2_
*t*_1_	0.04	0.04	0.04	0.03	0.10
*t*_2_	0.04	0.04	0.05	0.08	0.10

**Table 4:** Parameter estimates for *A. fulgidus* and *B. subtilis.* **Q** matrix parameters are scaled so that the expected number of events per unit time is 1, and edge lengths *t*_1_ (from the root to *A. fulgidus*) and *t*_2_ (from the root to *B. subtilis*) are in expected numbers of events per gene family. Parameters that were constrained to be zero are indicated by -. In the birth-death model, *h* was constrained to be *f* + *f*_2_.

model	blocks	blocks, d = 0	no multi-gene gains	no multi-gene losses	birth-death
log likelihood	−7.0311 × 10^3^	−7.0311×10^3^	−7.0379×10^3^	−7.0439×10^3^	−7.0683×10^3^
*a*	2.11	2.12	1.00	-	-
*b*	6.04×10^−7^	6.18×10^−7^	2.89×10^−7^	-	-
*b*_2_	0.24	0.23	0.09	-	-
*c*	7.00×10^−6^	9.29×10^−8^	-	1.93×10^−4^	-
*c*_2_	0.30	0.31	-	0.02	-
*d*	6.79×10^−8^	-	-	1.51×10^−4^	-
*e*	0.40	0.40	0.28	0.14	0.14
*f*	2.51×10^−3^	1.21×10^−4^	3.53×10^−5^	1.39	2.30
*f*_2_	3.57×10^−4^	3.49×10^−4^	3.02	1.84	−1.58
*g*	0.86	0.84	1.06	1.00	1.83
*g*_2_	0.03	0.07	1.08 × 10^−4^	2.25×10^−3^	−1.28
*h*	1.25	1.25	1.05	0.76	*f* + *f*_2_
*t*_1_	0.33	0.33	0.57	1.57	1.51
*t*_2_	0.52	0.52	0.69	1.11	1.51

**Table 5:** *E. coli* observed pattern counts. Element *i*, *j* is the number of gene families having state *i* in the K12 strain and state *j* in the 0157:H7 EDL933 strain. States (labelled in the first row and column) are 0, 1,… 19,≥ 20 family members.

	0	1	2	3	4	5	6	7	8	9	10	11	12	13	14	15	16	17	18	19	≥20
**0**	2622	79	21	5	3	3	0	0	2	3	1	0	1	1	0	0	0	0	0	0	1
**1**	55	1349	41	14	4	0	4	1	0	0	0	1	0	0	0	0	0	0	0	0	0
**2**	4	28	260	21	10	2	1	0	0	0	1	0	0	0	0	0	0	0	0	0	0
**3**	0	2	12	82	16	4	1	0	0	0	0	0	1	0	0	0	0	0	0	0	0
**4**	1	1	2	10	40	4	4	0	1	0	0	0	0	1	0	0	0	0	0	0	0
**5**	0	0	0	1	5	21	4	3	0	0	0	0	0	0	0	0	0	0	0	0	0
**6**	0	0	1	1	3	5	8	1	1	1	0	0	1	0	0	0	0	0	0	0	0
**7**	0	0	0	0	1	0	1	3	1	0	0	0	0	0	0	0	0	0	0	0	0
**8**	0	0	0	0	0	1	0	2	1	4	1	0	0	0	0	1	0	0	0	0	0
**9**	0	0	0	0	0	0	1	1	1	1	1	2	0	0	0	0	0	0	0	0	0
**10**	0	0	0	0	0	0	0	1	0	2	0	1	0	1	0	0	0	0	0	0	0
**11**	1	0	0	0	0	0	0	0	0	0	0	0	1	1	0	0	1	0	0	0	0
**12**	0	0	0	0	0	0	0	0	0	0	3	0	2	1	0	0	0	0	0	0	1
**13**	0	0	0	0	0	0	0	0	1	0	0	0	0	2	0	0	0	0	0	0	0
**14**	0	0	0	0	0	0	0	0	0	0	0	0	0	0	0	0	0	0	0	0	1
**15**	0	0	0	0	0	0	0	0	0	0	0	0	0	0	1	2	0	0	0	0	0
**16**	0	0	0	0	0	0	0	0	0	0	0	0	0	0	0	0	0	0	0	0	0
**17**	0	0	0	0	0	0	0	0	0	0	0	0	0	0	0	0	0	0	1	0	0
**18**	0	0	0	0	0	0	0	0	0	0	0	0	0	0	0	0	0	0	0	0	1
**19**	0	0	0	0	0	0	0	0	0	0	0	0	0	0	0	0	0	0	0	1	0
**≥20**	0	0	0	0	0	0	0	0	0	0	0	0	0	0	0	0	0	0	0	0	5

**Table 6:** *Archaeoglobus fulgidus* and *Bacillus subtilis* observed pattern counts. Element *i*, *j* is the number of gene families having state *i* in *A. fulgidus* and *state j* in *B. subtilis.* States (labelled in the first row and column) are 0, 1,…19, ≥ 20 family members.

	0	1	2	3	4	5	6	7	8	9	10	11	12	13	14	15	16	17	18	19	≥20
**0**	2448	857	180	66	28	12	11	8	5	6	2	1	1	1	1	0	1	0	0	0	1
**1**	509	284	87	29	12	3	1	0	2	0	0	1	0	0	0	0	0	0	0	1	3
**2**	86	43	26	13	4	2	1	2	1	1	1	0	0	0	1	0	0	0	0	0	0
**3**	22	12	1	3	1	0	0	2	2	0	0	0	0	0	0	0	0	1	0	0	0
**4**	18	7	4	2	2	1	1	0	1	0	0	0	0	0	0	1	0	0	0	0	1
**5**	6	2	0	2	0	0	0	0	0	0	0	0	0	0	0	0	0	0	0	0	0
**6**	6	1	0	0	0	0	0	0	0	0	0	0	0	0	1	0	0	0	0	0	0
**7**	1	0	0	0	1	0	0	0	0	0	0	1	0	0	1	0	0	0	0	0	0
**8**	0	0	2	0	0	0	0	1	0	0	0	0	1	1	0	0	0	0	0	0	0
**9**	1	0	0	0	0	0	2	0	0	1	0	0	0	0	0	0	0	0	0	0	0
**10**	0	0	1	0	0	0	0	0	0	0	0	0	0	0	0	0	0	0	0	0	0
**11**	3	0	0	0	0	1	0	1	0	0	0	0	0	0	0	0	0	0	0	0	0
**12**	1	0	0	1	0	0	0	0	0	0	0	0	0	0	0	0	0	0	0	0	0
**13**	0	1	0	0	0	0	0	0	1	0	0	0	0	0	0	0	0	0	0	0	0
**14**	0	0	0	0	0	1	0	1	0	0	0	0	0	0	0	0	0	0	0	0	1
**15**	0	0	0	1	0	0	0	0	0	0	0	0	0	0	0	0	0	0	0	0	0
**16**	0	0	0	0	0	0	0	1	0	0	0	0	0	0	0	0	0	0	0	0	0
**17**	1	0	0	0	0	0	0	0	0	0	0	0	0	0	0	0	0	0	0	0	0
**18**	0	0	0	0	0	0	0	0	0	0	0	0	0	0	0	0	0	0	0	0	0
**19**	0	0	0	0	0	0	0	0	0	0	0	0	0	0	0	0	0	0	0	0	0
**≥20**	0	0	0	0	0	0	0	0	0	1	0	0	0	0	0	0	0	0	1	0	0

**Table 7:** Rate matrix **Q***_X_* for the Markov chain used in calculating expected residence time of a gene (Section ). Here, *k* = 3. The *q_ij_* are from the original Markov chain, and **Q***_I_* is rows and columns 0 to ≥ 3 of Q*_X_*. Diagonal elements (not shown) are −1 times the row sums of off-diagonal elements in **Q***_X_*.

	0	1	2	3	χ
0	-	q_01_	*q*_02_	*q*_03_	0
1	*q*_10_α_10_	-	*q*_12_	*q*_13_	*q*_10_(1 − α_10_)
2	*q*_20_α_20_	*q*_21_α_21_	-	*q*_23_	*q*_20_(1 − α_20_) + *q*_21_(1 − α_21_)
3	*q*_30_α_30_	*q*_31_α_31_	*q*_32_α_32_	-	*q*_30_(1 − α_30_) + *q*_31_(1 − α_31_) + *q*_32_(1 − α_32_)
χ	0	0	0	0	-

## Appendix

### Appendix: methods

#### Data

The COG (Clusters of Orthologous Groups of proteins) database (Tatusov et al. 2003) contains data on the number of members of each of 4873 gene families in each of 66 taxa, of which 63 are prokaryotes (downloaded 13 May 2004 from ftp://ftp.ncbi.nih.gov/pub/COG/). Gene family recognition is based on patterns in pairwise sequence similarity detected using BLAST (Tatusov et al. 1997). Because of the way this is done, a family will not appear in the database unless it is present in at least three taxa. We therefore need to make some correction for unobservable families. We extrapolated from the relationship between the number of observations of each pattern of presence and absence of gene families in a pair of genomes and the number of genomes in which a gene family occurs, using locally-weighted least squares (LOWESS) as described below. We then used the predicted numbers of unobserved instances to form LOWESS imputed counts *n̂**_kl_* for each pattern, where *k* and *l* are the number of members of a gene family in each of two genomes. We based all subsequent calculations on the imputed counts.

To develop the models described here, we worked mainly with two pairs of taxa. The two *E. coli* strains K12 and 0157:H7 EDL933 are closely related, having different gene content for 433 out of 4873 families. The non-pathogenic K12 has 2131 families present, of which 4 have more than 20 members and none has more than 61 members. The pathogen 0157:H7 EDL933 has 2190 families present, of which 8 have more than 20 members and none has more than 62 members. Table 5 shows the observed numbers of families with *i* members in K12 and *j* members in 0157:H7 EDL933. The largest count is for families that are absent in both strains. Most families have small and similar numbers of members in each strain. *Archaeoglobus fulgidus* (archaea) and *Bacillus subtilis* (gram positive bacteria) are distantly related, having different gene content for 2109 out of 4873 families. *A. fulgidus* has 1244 families, of which 2 have more than 20 members and none has more than 25 members. *B. subtilis* has 1771 families, of which 5 have more than 20 members and none has more than 58 members. Table 6 shows the observed numbers of families with *i* members in *A. fulgidus* and *j* members in *B. subtilis*. Compared to the *E. coli* data, there are fewer families with large numbers of members in both taxa, and more with several members in one taxon and none in the other.

#### Correcting for unobservable data

In the COG database, gene families that are present in fewer than three genomes are not observable. In order to compute likelihoods, we need to correct for the unobservable data.

One method is to consider only the gene families present in some reference genome or set of genomes (Lake and Rivera, 2004). We did not use this method because it is not clear how we should choose a reference genome or set of genomes that is sufficiently independent of the taxa under study. For example, we might expect the set of all genomes in COG to be a reasonable reference choice. Nevertheless, we found strong dependencies between the presence/absence of gene families in pairs of taxa and the number of genomes in which those families occur in the entire database (results not shown). On average, gene families absent from a pair of taxa tend to be found in few genomes, so conditioning on all COG genomes would not be appropriate.

Another approach is to work with likelihoods conditional on the family appearing in the database (Gu, 2000). Conditional likelihoods are difficult to apply to pairwise analyses of the COG database. A family could be absent from both members of a pair, yet still appear in the database if it is present in some other taxon. Since absence from both members of a pair is by far the most common pattern, discarding all double absences (Gu and Zhang, 2004) does not make good use of the available data. Furthermore, a family could be present in both members of a pair, yet still be absent from the COG database. Conditional likelihoods would be a good choice for an analysis of the entire database, although this would be time-consuming because we would have to calculate the probabilities of a family being absent from all genomes, present in only one genome (for all choices of one genome), and present in only two genomes (for all choices of two genomes). These probabilities are dependent on the tree structure and the model parameters. We could reduce the computing time by extending the database to include gene families that occur in only one or two genomes (Boussau et al. 2004), although it is not possible to avoid an arbitrary choice of significance threshold for pairwise sequence similarity, which makes it hard to analyze closely- and distantly-related genomes at the same time (Tatusov et al. 1997).

Instead, we estimated the numbers of unobserved instances of each pattern of *k* members of a gene family in one genome and *l* members in the other. For each pair of genomes, we extrapolated from the relationship between the number of observations of each presence-absence pattern and the number of genomes in which a gene family occurs using locally-weighted least-squares (LOWESS). We then used the predicted numbers of unobserved instances to form LOWESS imputed counts *n̂**_kl_* for each pattern of *k* members of a gene family in one genome and *l* members in the other.

#### LOWESS extrapolation

The data for the focal pair of taxa are the number of observations *n**_kl_* of each pattern for a pair of genomes. Most patterns have few observations, so we aggregate patterns into the categories *AA, AP, PA* and *PP,* where *A* means absent and *P* means present. We have the number of observations *n**_AA_*(*n**_g_*), *n**_AP_*(*n**_g_*), *n**_PA_*(*n**_g_*), *n**_PP_* (*n**_g_*) of each category in the pair of genomes for families detected in *n**_g_* = 3,4,…,*G* genomes, where *G* is the number of genomes in the database. A family absent from both genomes in the focal pair could be present in 0, 1 or 2 genomes in the whole database without being detected. For the category *AA*, we therefore want to predict the number of occurrences *n**_AA_*(*n**_g_*) for *n**_g_* = 0,1,2. For categories *AP* and *PA,* the family must be present in at least one genome in the database (one member of the focal pair), so we want to predict the number of occurrences for *n**_g_* = 1,2. For *PP*, the family must be present in at least two genomes in the database (the two genomes in the focal pair), so we want to predict the number of occurrences for *n**_g_* = 2.

Figure 6 shows the relationship between *n**_g_* and the number of observations of each category for the pairs *E. coli* strains K12 and 0157:H7 EDL933 (Figure 6a, c, e, g) and *Archaeoglobus fulgidus* and *Bacillus subtilis* (Figure 6b, d, f, h). For the *AA* pattern (Figure 6a and b), there is a strong curvilinear relationship. For the other categories, the relationships do not suggest any simple parametric form, and differ between the two pairs. For example, the *E. coli* pair has *n**_PP_*(*n**_g_*) (Figure 6g) increasing rapidly as *n**_g_* increases from 3 to 8, then decreasing slowly. There is a large outlier at *n**_g_* = 66 (many gene families present in both *E. coli* strains are also present in every other genome in the database). For *A. fulgidus* and *B. subtilis*, there is a much more gradual increase in *n**_PP_* (*n**_g_*) (Figure 6h) with *n**_g_*_,_ again with a large outlier at *n**_g_* = 66. We have no theory to guide the choice of model. We therefore used LOWESS with linear local models, tricube weights, and the span (proportion of data to include in local regressions) chosen by leave-one-out cross-validation (Cleveland, 1979). We set negative predicted values to zero. The fitted models generally behave well (Figure 6). The *n**_PP_* (*n**_g_*) case for *E. coli* (Figure 6g) is not very smooth, which makes extrapolation to *n**_g_* = 2 potentially unreliable, but in this case the number of occurrences of *PP* will be small for any reasonable model. In both cases, by far the largest number of unobserved events are *AA.*

We examined plots of the LOWESS fits for all pairs of taxa. The only cases where the LOWESS predictions did not closely match the observed counts for small *n**_g_* were where the first member of the pair was one of the three eukaryotes in the database (*Saccharomyces cerevisiae, Schizosaccharomyces pombe* and *Encephalitozoon cuniculi*), and the other member of the pair was not a eukaryote. For these cases, *n**_PA_*(3) was always much larger than expected, because there are many genes present in all three eukaryotes and no other genomes. The optimal spans for these cases were large, because the other data were smooth, so the LOWESS curves did not pick up the outliers.

#### Imputed counts

Given models for each category, we obtain the predicted counts *n̂**_AA_*(*n**_g_*), *n̂**_AP_* (*n**_g_*), *n̂**_PA_*(*n**_g_*), *n̂**_PP_* (*n**_g_*) at all values of *n**_g_*. The predicted proportions of each category are

(4) $${\hat{p}}_{g(kl)}=\sum_{n_{g}}{\hat{n}}_{g(kl)}/\sum \hat{n}$$

where ∑*n̂* is the sum of predicted counts over all categories and values of *n**_g_* and

(5) $$g\;(kl)=\{\begin{array}{ll} AA, & k=0,l=0\\ AP, & k=0,l>0\\ PA, & k>0,l=0\\ PP, & k>0,l>0 \end{array}$$

The imputed count *n̂**_kl_* including unobserved families, is then

(6) $${\hat{n}}_{kl}={\hat{p}}_{g\;(kl)}Nn_{kl}/\sum_{ij|g\;(ij)=g\;(kl)}n_{ij}$$

where *N* = ∑*_ij_* *n**_ij_* is the sum of observed counts (the total number of gene families in the database). The (0, 0) pattern is the only one in the *AA* category, so the imputed count is just the predicted proportion of the category times the total number of observed gene families. The other categories each contain many patterns. We therefore estimate the imputed count *n̂**_kl_* for a pattern in one of these categories as the predicted proportion of the category times the total number of observed gene families times the proportion of the original data for the category contributed by the pattern.

The (0,0) pattern is higher in the imputed counts than in the original data for both pairs of taxa (Figure 7, upper right-hand point). All other patterns have their counts decreased by roughly the same proportion.

#### Parameter estimation and model comparison

For each model, we found the maximum likelihood parameter estimates numerically using Matlab Release 14 (The Mathworks, Inc, Natick, MA). For both models, we estimate all **Q** matrix parameters relative to the rate *e* of transitions from state 0 to state 1. A legitimate **Q** matrix has strictly nonnegative off-diagonal entries. For elements of the form $\left(\begin{array}{c} i\\ \left|j-i\right| \end{array}\right)x+x_{2}$ (where *x* and *x*_2_ are the linear and constant components of some process such as duplication or deletion) we require the linear component to be non-negative because we expect the rate of events not to decrease with the number of units on which the events can operate. Then *x*_2_ > −*x* will always ensure the entries are nonnegative. We constrained all parameters to be non-negative (using a log transformation) unless the *x*_2_ estimates were very close to zero. If this occurred, we iteratively re-estimated parameters, setting the lower bound for the relevant *x*_2_ to −*x* and re-fitting until the estimate was no longer on the boundary. For the more complex models, we obtained different log likelihoods for different initial conditions due to local minima in the likelihood function. We therefore used the best of 10 estimates from random initial conditions for each of the two pairs of taxa.

Including edge lengths, the blocks model has 13 free parameters and the birth-death model has five. Although there are 14 parameters in the blocks model, one is fixed because we scale the **Q** matrix so the expected number of events per unit time is one. In the birth-death model, the two edge lengths count as a single parameter because the likelihood is affected only by their sum. We can obtain the birth-death model from the blocks model by setting *a, b, b*_2_*, c, c*_2_ and *d* to zero, and *h* to *f* +*f*_2_. We can therefore use a likelihood ratio test to compare these models. In standard cases, the asymptotic distribution of twice the log likelihood ratio (−2Δlog *L*) when the simpler model is true is *χ**_n_*^2^ for nested models, where *n* is the difference in number of parameters between the two models (in this case 8). This does not hold for the comparison between the birth-death and blocks models, because the parameters *a, b, b*_2_*, c, c*_2_ and *d* are being set to boundary values (Self and Liang, 1987). Nevertheless, the standard *χ*_8_^2^ test will usually be conservative (Ota et al. 2000) (and appeared to be so in test cases where we ran simulations). Similarly, we used a conservative *χ*_3_^2^ test to evaluate blocks models with no multiple gene losses (*a*, *b* and *b*_2_ set to zero) or no multiple gene gains (*c*, *c*_2_ and *d* set to zero).

Each model is associated with a set of predicted relative pattern frequencies *f̂**_ij_* (model) the proportion of gene families that will be in state *i* in species 1 and state *j* in species 2. These are

(7) $$F={\left(e^{Qt_{1}}\right)}^{\text{T}}S\;(e^{Qt_{2}})$$

where **F** is the matrix of pattern frequencies, **S** is a matrix with the stationary probabilities π_i_ along the diagonal, and ^T^ indicates transpose. The log likelihood of a model can be written as ∑*_ij_* *n̂**_ij_*(log *f̂**_ij_*_(model)_, where *n̂**_ij_* is the LOWESS imputed count of state *i* in species 1 and state *j* in species 2. The best possible model is one that reproduces the LOWESS imputed frequencies *n̂**_ij_*/*n* exactly. A natural way to determine where the model fits well and where it fits badly is to plot the contribution to the log likelihood ratio for the comparison between a given model and the best possible model from each pattern, *n̂**_ij_*(log *f̂**_ij_*_(model)_ − log(*n̂**_ij_*−*n*)). This is positive when the model predicts a higher frequency than observed, negative when the model predicts a lower frequency than observed, and sums over all *i* and *j* to the log likelihood ratio. We will do this for the blocks and birth-death models.

We also want to test whether the rates of lateral transfer are greater than zero. Rejecting the hypothesis that *d* is zero is evidence for a non-zero rate of lateral transfer rate of multiple genes from the same family. Because *d* = 0 is a boundary value, the asymptotic distribution of −2Δ log *L* for this test is $\frac{1}{2}\chi_{0}^{2}+\frac{1}{2}\chi_{1}^{2}$ (Self and Liang, 1987) when compared to a blocks model with unrestricted *d*. Although other transitions may involve lateral transfer, we cannot use them to estimate lateral transfer rates. The transition from state 0 to state 1 may occur either by innovation or by lateral transfer. We might expect innovation (evolution of a new member of a gene family from some other sequence) to be a very rare event, in which case the rate *e* will be almost entirely due to lateral transfers. Nevertheless, we cannot separate the two components of *e.* Furthermore, testing whether *e* is greater than zero is not sensible, because if *e* is zero, the stationary distribution has all its mass in state zero, and any pattern other than the absence of a family in every taxon has likelihood zero. We have no way to separate the lateral transfer, innovation and duplication components of transitions from state *i* to states *i* < *j* ≤ 2*i*. Thus, we cannot say anything about the importance of lateral transfer in these transitions.

#### Expected residence times for genes

Given a **Q** matrix, we can calculate the expected residence time *E*(*r*) for a gene. This is the time interval from the appearance of a gene in a genome (by duplication, lateral transfer or innovation) to the deletion of this gene:

(8) $$E(r)=\sum_{i=0}^{k}\beta_{i}r_{i}$$

where β_i_ is the probability that we enter state *i* as a gene appears in the genome, and *r**_i_* is the expected time until a gene is deleted, given that we were in state *i* when it appeared in the genome. At steady state,

(9) $$\beta_{i}=\sum_{j<i}q_{ji}\;\pi_{j}(i-j)/\sum_{i}\sum_{j<i}q_{ji}\;\pi_{j}(i-j)$$

The numerator is the sum of steady-state rates of flow into state *i* that add new genes, weighted by the number of genes *i* − *j* each flow adds. The denominator normalizes the β*_i_* to probabilities.

To calculate the *r**_i_*, we construct a new Markov chain with one extra state *X*, that represents loss of a given gene. We assume that all members of a family are equally likely to be deleted. Then during the transition from having *i* to *j* members of a family where *j* < *i*, the probability that a focal gene will not be among those deleted is α*_ij_* = *j*/*i*. If the given gene is deleted, with probability 1 − α*_ij_**,* we enter state *X*. The *r**_i_* are then the expected hitting times for state *X*, starting from state *i*. These are the solutions to the linear equations

(10) $$-Q_{I}r=1$$

(Norris, 1997, p. 113), where **Q***_I_* is rows and columns *i* = 0,1…*k* of the rate matrix **Q***_X_* for the new Markov chain (ie all rows and columns except those for state *X*), **r** is a column vector of residence times *r**_i_*, and **1** is a column vector of *k* + 1 ones. Table 7 shows an example of **Q***_X_* and **Q***_I_*.

We calculated expected residence time (measured in expected numbers of events) under the blocks model. We also ran Monte Carlo simulations to determine the distribution of residence times. We initialized a simulated genome in a state *i* sampled from the stationary probability for the **Q** matrix. We sampled the time to the next event from the exponential distribution with parameter −*q**_ii_* (the rate of leaving the current state), and sampled the new state of the genome from the multinomial with parameters given by the off-diagonal entries in the *i*th column of **Q**. If the transition involved a deletion of some genes, we deleted each gene with equal probability. We recorded the time at which each gene was created and deleted, and ran the simulation until we had obtained the residence times of 10000 genes, after allowing 10000 simulated residence times to reach stationarity.

#### Phylogeny for genomes in the COG database

Full maximum likelihood estimation of model parameters and a tree topology with edge lengths is not feasible in our current implementation. We therefore fitted the blocks model and the birthdeath model to all 2145 pairs of taxa from the COG database, and used the resulting estimates of evolutionary distance to construct a phylogeny based on gene content. Some pairs were much more difficult to optimize than the *E. coli* and *A. fulgidus*/*B. subtilis* pairs we examined in detail, having many local minima in the likelihood surface for the blocks model. We therefore selected the best from 30 sets of random initial conditions for each pair. If the best fitting blocks model had a lower likelihood than the best birth-death model, we know that the blocks model optimization failed, because the birth-death model is nested within the blocks model and should always have lower likelihood. In these cases, we re-optimized the blocks model, starting close to the birth-death parameter estimates. Such problems occurred in 38 out of 2145 pairs, all but one of which involved an archaeon or a eukaryote being compared with a bacterium. The fitting took around 3 weeks on an Intel Xeon 3.06 ghz processor with 4 gigabytes of RAM (this time requirement meant that bootstrapping was not feasible). We then used the sums of edge lengths (*t*_1_ +*t*_2_) for each pair from each model as maximum likelihood estimates of evolutionary distance. We used PAUP* version 4b10 for UNIX (Swofford, 2003) to perform heuristic tree-bisection-reconnection searches for the best weighted least-squares trees, starting from initial neighbor-joining trees. The weights were the reciprocals of squared distances, and edge lengths were constrained to be non-negative.
